# Two Is Better than One: Successful World-Class Sprinters Compete in Two Disciplines

**DOI:** 10.3390/jfmk8020052

**Published:** 2023-04-27

**Authors:** Paolo Riccardo Brustio, Alberto Rainoldi, Gennaro Boccia

**Affiliations:** 1 Department of Clinical and Biological Sciences, University of Turin, 10126 Turin, Italy; 2 NeuroMuscularFunction Research Group, School of Exercise & Sport Sciences, University of Turin, 10126 Turin, Italy; 3 Department of Medical Science, University of Turin, 10126 Turin, Italy

**Keywords:** performance progression, career trajectories, specialization, track and field

## Abstract

We aimed to quantify the prevalence of track and field sprinters competing at a world-class level in more than one discipline, and we describe the career characteristics of single- and double-discipline athletes in terms of peak performance and the age of peak performance. The career performance of athletes ranked in the top 200 positions of the World Athletics database in the 100 m, 200 m, and 400 m were analyzed, i.e., 5514 career profiles (49.9% female). Using binomial proportion, we calculated how many competed in only one or more than one discipline. We also compared the peak performance and the age of peak performance of athletes who competed in one vs. more than one discipline. Independent of gender, about 50% of the athletes competing in the 100 m and 200 m also competed in the other discipline (i.e., 200 m and 100 m, respectively). Differently, only 20% of the athletes competing in the 400 m also competed in the 200 m. Sprinters competing in the 100–200 m and 200–400 m couples showed better peak performance than the sprinters competing in only one discipline. Many world-class sprinters compete in two disciplines, and the 100–200 m was the most prevalent couple. Our results also suggest that sprinters who compete in two disciplines may be advantaged compared to sprinters who compete in only one event.

## 1. Introduction

Various studies on track and field disciplines have provided information about the career development of athletes [1,2,3,4,5,6,7]. In the international arena, longitudinal assessments of athlete career trajectories may provide useful data, including peak performance age. Knowing the typical age of peak performance is crucial to assist athletes, coaches, and federations in determining realistic long-term performance goals. Hollings et al. [8] studied the career performances of the top 12–16 athletes at the Olympic Games or World Athletics Championships between 2000 and 2009. They estimated a mean range age of peak performance from 24.5 (100 m and 400 m) to 25.0 years (200 m) in males and from 24.8 (400 m) to 25.4 (100 m) years in female sprinters, with a similar peak performance window (~3–4 years). Interestingly, the age of peak performance in explosive events, such as sprint events, tended to decrease with the sprinting distance increasing (from 100 to 400 m) [8,9].

Most previous studies have overlooked that an athlete’s youth career may affect reaching their peak performance in adulthood [3,4,5,10,11,12]. In this regard, we found that sprinters performing exceptionally in their youth career reached their peak performance earlier (overall mean: 20.1 and 19.8 years for males and females, respectively) than successful sprinters both in their youth and senior careers (overall mean: 22.3 and 23.2 years for males and females, respectively) or that they reached success only during their senior career (overall mean: 24.3 and 25.1 years for male and female sprinters, respectively) [2]. These data suggest that athletes who start young may also reach their peak performance earlier than their counterparts who specialize somewhat later [2,13].

One of the most apparent simplifications of our [2] and other investigations [8,9] was that we separately analyzed the individual sprinter disciplines. While this simplification may help identify the benchmarks (e.g., the window of peak performance) for success in each discipline, it does not represent the reality of sprinter competitions. Indeed, many sprinters compete in multiple sprint disciplines, with the 100–200 m and 200–400 m couples likely the most common ones. Nevertheless, to the best of our knowledge, no study has investigated if the career trajectories of world-class sprinters are affected by competing in more than one discipline during their career. Considering if an athlete is competing (at a significant level) in more than one discipline is considerable to interpret performance progression. Reporting the typical age of peak performance and differentiating the athletes competing in one to two disciplines would be informative for coaches to understand athletes’ career progression better.

Therefore, in this first attempt, we aimed to (1) describe the prevalence of world-class athletes able to successfully compete in more than one sprinter discipline (i.e., the 100–200 m and 200–400 m couples) and (2) describe the benchmarks of performance in terms of the peak and peak age performance of sprinters who competed in one vs. two sprinter disciplines.

## 2. Materials and Methods

This study further analyzed the data collected in previous publications [2,14]. Here, we maintained the same databases but rethought the analysis with different research questions. The names of male and female world-class sprinters (i.e., 100 m, 200 m, and 400 m) ranked in the top 100 official lists of World Athletics (from 2000 to 2018) and/or participated in the World U18 or U20 Championships (from 1998 to 2015) were collected. After merging the two databases and removing all duplicate names, each sprinter’s annual best performance and the date of the annual best performance were downloaded and included in the primary dataset. If the athlete was still competing, the annual best performances were downloaded from the first to last appearance in the World Athletics database on 31 December 2018. Only results recorded in outdoor competitions and with regular wind were considered. Athletes disqualified for doping offences were excluded from the analysis (i.e., 112 athletes, 50.0% female). All the data were available in the public database of World Athletics (https://www.worldathletics.org/, last accessed on 3 December 2018), and thus, no informed consent was obtained. Ethical review and approval were not obtained for this study because the data are available online.

## 3. Procedure

Only sprinters who competed at least once in the senior category (i.e., from 20 years) were considered. All annual performances were normalized to the corresponding discipline and gender World Record. Subsequently, separately for each discipline and gender, the sprinters were ranked according to their best normalized annual performance in the all-time ranking. Only sprinters in the top 200 all-time rankings entered the final database.

Subsequently, the sprinters were divided into five subgroups as follows:

- *Only 100 m:* corresponding to the sprinters who only competed in the 100 m discipline.
- *Only 200 m:* corresponding to the sprinters who only competed in the 200 m discipline.
- *Only 400 m:* corresponding to the sprinters who only competed in the 400 m discipline.
- *100 and 200 m:* corresponding to the sprinters competing in the 100 m and 200 m disciplines.
- *200 and 400 m*: corresponding to the sprinters competing in the 200 m and 400 m disciplines.

Due to the low number of athletes who competed in the 100 m and 400 m and 100 m, 200 m, and 400 m, these categories were not included in this analysis. The peak performance and the age of peak performance characterizing the career of sprinters were calculated. All of the sprinter data were extrapolated using custom-written software in MATLAB R2020b (MathWorks, Natick, MA, USA).

### 3.1. Statistical Analysis

Using the binomial proportion confidence interval [90% CI], the proportions of sprinters who competed in the 100 m (i.e., the only 100 m and 100 and 200 m subcategories), the 200 m (i.e., the only 200 m, 100 and 200 m, and 200 and 400 m subcategories) and the 400 m (i.e., the only 400 m and 200 and 400 m subcategories) were calculated.

Then, to compare the peak performance and the age of peak performance among sprinters competing only in the 100 m, 200 m, and 400 m (i.e., the only 100 m, only 200 m and only 400 m subcategories) or in the 100–200 m and 200–400 m couples (i.e., the 100 and 200 m and 200 and 400 m subcategories) a one-way ANOVA was carried out. The Welch’s F test was performed when the homogeneity of variances was violated (i.e., *p* < 0.05 Levene’s test of homogeneity of variance). Bonferroni post hoc and Games–Howell post hoc corrections were used to identify specific subgroup differences in sprinters competing in the 200 m.

All of the analyses were separately performed for gender. All of the data were analyzed using MATLAB R2020b (Mathworks, Natick, MA, USA) and JASP Team (Version 0.12). The chord diagrams were prepared by an online tool (http://www.datasmith.org/2018/06/02/a-bold-chord-diagram-generator/, last accessed on 4 February 2023). The level of significance was set at *p* ≤ 0.05.

### 3.2. Results

**Figure 1:**
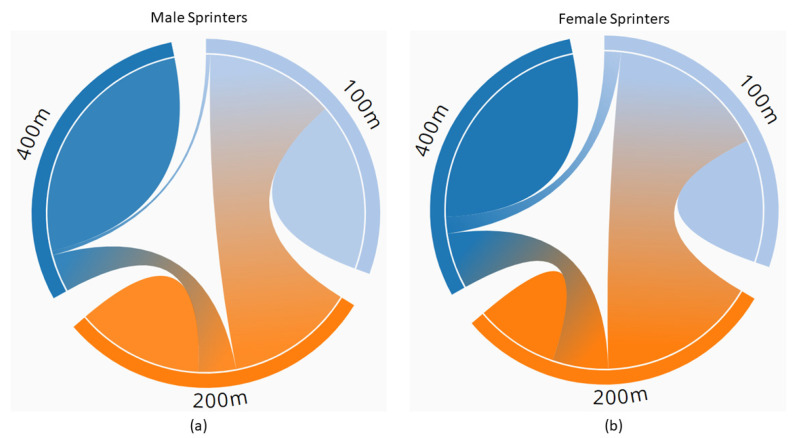
This figure represents the chord diagrams of the proportion of male (**a**) and female (**b**) athletes who competed in only one or more than one discipline.

The career profiles of 5514 (49.9% female) with at least one annual best performance during their senior career were evaluated. One thousand two hundred and eleven sprinters (50.1% female) met the inclusion criteria and were included in the analysis. Figure 1 represents the chord diagrams of the proportion of male (a) and female (b) athletes who competed in only one or more than one discipline.

*100 m.* Considering the male sprinters who competed in the 100 m, 55.8% (90% CI [49.8, 61.6]) of them competed only in the 100 m, 42.8% [37.0, 48.7] competed in the 100 m and 200 m, and 1.4% [0.4–3.7] competed in the 100 and 400 m. For the female sprinters, the prevalence was 41.1% [35.5, 47.0], 53.3% [47.4, 59.1], and 5.6% [3.3, 8.9] for the only 100 m, 100 and 200 m, and 100 and 400 m subgroups, respectively.

*200 m.* Considering the male sprinters who competed in the 200 m, 43.3% [37.5, 49.4] of them competed only in the 200 m, 43.8% [38.0, 49.9] competed in the 100 m and 200 m, and 12.8% [9.1, 17.3] competed in the 200 m and 400 m. For the female sprinters, the prevalence was 28.0% [23.0, 33.5], 53.3% [47.4, 59.1], and 18.7% [14.4, 23.6] for the only 200 m, 100 and 200 m, and 200 and400 m subgroups, respectively.

*400 m.* Finally, considering the male sprinters who competed in the 400 m, 85.7% [81.0, 89.6] of them competed only in the 400 m, 12.8% [9.1, 17.3] competed in the 200 m and 400 m, and 1.5% [0.4, 3.8] competed in the 100 and 400 m. For the female sprinters, the prevalence was 75.4% [70.0, 80.2], 19% [14.6, 24.0], and 5.7% [3.3, 9.1] for the only 400 m, 200 and 400 m, and 100 and 400 m subgroups, respectively. The data on the peak performance and the age of peak performance are presented in Table 1.

Most of the results showed that the athletes who competed in two disciplines performed better than those who competed in only one discipline. For example, in male sprinters, the 100 and 200 subgroup showed better 200 m peak performance than the only 200 m subgroup. Similarly, the 200 and 400 m subgroup showed better 400 m peak performance than the only 400 m subgroup. In the female sprinters, the 100 and 200 m subgroup showed better 100 m and 200 m peak performance than the only 100 m and only 200 m subgroups, respectively. Furthermore, the 200 and 400 m subgroup showed better 200 m and 400 m peak performance than the only 200 m and only 400 m subgroups, respectively.

The age of peak performance did not vary across the subgroups in the males, whereas in the females, the only 200 m subgroup showed an earlier age of peak performance (~23 years) compared to the 100 and 200 m (~24 years) and 200 and 400 m (~25 years) subgroups.

## 4. Discussion

We aimed to quantify the prevalence of track and field sprinters competing at a world-class level in one or more disciplines, and we have described the career characteristics of single- and double-discipline athletes in terms of peak performance and the age of peak performance. The main findings of the present study are: (1) approximately 50% of the sprinters competing in the 100 m and 200 m were also highly competitive in the other discipline (i.e., 200 m and 100 m, respectively); (2) ~80% of the sprinters competing in the 400 m competed only in this discipline and were not highly competitive in the 200 m; and (3) the sprinters competing in the 100–200 m and 200–400 m couples showed slightly better peak performance than the sprinters competing in only one discipline.

The most expected finding of the present study was that among the athletes who competed in more than one discipline, the 100–200 m couple was about three times more frequent (114 athletes) compared to the 200–400 m couple (40 athletes). In other words, 40–50% of athletes competing in the 100 m were highly competitive in the 200 m and vice versa, whereas only 20% of athletes competing in the 200 m were also competitive in the 400 m and vice versa. Even if sprint performance in the three different distances is determined primarily by common factors (i.e., reaction time, acceleration, maximum running velocity, and the ability to sustain this in increasing fatigue) [15], evident differences also exist. The different aerobic–anaerobic energy system contributions in the three distances may explain the similarity between 100 and 200 m. Indeed, while in the 100 and 200 m races, the relative aerobic–anaerobic energy contribution ranges between 20–30% and 70–80%, respectively [16], the aerobic–anaerobic energy system contribution is about 40% and 60%, respectively [17]. Thus, our findings confirm that practising for improving different interactions in metabolic pathways supply (100–200 vs. 400 m) can be challenging, and for this reason, very few sprinters can successfully perform all three different distances. Moreover, differences in running mechanics over 100 m, 200 m, and 400 m may contribute to these results [18]. The average speed is about 10 m/s and is similar between 100 and 200 m, while for 400 m it is about 9 m/s (Table 1). Thus, it is possible to speculate that the 100 m and 200 m may be more similar in training and competition, while the 400 m requires more specific preparation and specialization [13].

Female sprinters tend to be more frequently specialized in two disciplines than males. For example, while ~50% of female sprinters who competed in the 100 m were highly competitive in the 200 m and vice versa, the proportion of male sprinters was only ~40%. While the present study was not designed to investigate gender differences, the results may suggest that it is easier for women to compete at a world-class level in more than one discipline. Again, as this is an observational study based solely on performance measures, we cannot speculate on the reasons for this trend. However, the present results may suggest that those female athletes could be afraid to prepare/compete in more than two disciplines, possibly increasing their chances of success in either one or two.

The most interesting and unexpected finding of the present study was that the subgroup of athletes competing in two disciplines (i.e., the 100–200 m and 200–400 m couples) showed slightly better performance compared to those who competed only in one discipline (Table 1), suggesting that multi-specialization, also during an athlete’s senior career, may confer an advance in terms of performance. This result can be explained in at least two different ways. First, we can speculate that competing and training in two disciplines may confer an advantage as opposed to over-focusing on one discipline. For example, the physical qualities trained for one discipline may support the development of the qualities needed for the other. Second, the athletic level of some athletes is higher than others, which may allow for competing in more than one discipline successfully. With the present data, we cannot disentangle the direction of this association. Are there superior physical qualities of some athletes that make them competitive in two disciplines, or is it the training for two disciplines that makes them so competitive? Given the observational nature of this study, we could not provide an answer, but future studies should try to understand if competing in more than one discipline during an athlete’s senior career may confer an advantage.

The data on the age of peak performance showed a similar trend to a previous study [8] with a range age of peak performance of approximately 24–25 years in male and female sprinters. Except for female sprinters competing in the 200 and 400 m, the age of peak performance did not vary across the subgroups. This means that competing in more than one discipline does not influence the age at which the peak performance is obtained. This was unexpected as we hypothesized that it would take more time to specialize in more than one discipline. Nevertheless, when analysing the age of peak performance, it is necessary to also consider timing specialization [13]. Therefore, as we analyzed senior athletes, specialization timing may affect the junior career more than the senior career.

The study has some limitations that should be underlined. Our analysis was based on annual performance. Thus, no other factors that may affect the results of the present study were considered, such as individuals’ maturity status or socio-cultural context affecting the selection and deselection process. Again, we considered only the top 200 athletes in the ranking; it is possible that the increase or the decrease in performance level may lead to different results. Finally, even if we normalized the annual performance to the corresponding discipline and gender World Record, the different decades of competition may have influenced the athletes’ ability to compete in one or more disciplines.

## 5. Practical Applications

The present study poses two main practical applications. The first perspective relates to future scientific research on athletes’ career paths: virtually all studies in the literature report the career characteristics of track and field athletes separately for each discipline. Here, we have shown that many world-class athletes compete in two disciplines. Consequently, the parameters adopted to characterize the career, with the age of peak performance being the most important, are likely interrelated across disciplines and should be treated accordingly. Second, the present findings suggest that sprinters competing in two disciplines may be advantaged compared to sprinters who compete in only one event. This may support the idea of multi-specialization: sprinters can/should specialize in more than one discipline during their adult careers. However, this suggestion should be supported by further analysis.

## 6. Conclusions

The career performance of the all-time best 200 sprinters competing in the 100 m, 200 m, or 400 m disciplines has been tracked. The main findings suggest that it is possible, and possibly even favourable, for world-class athletes to compete in more than one sprint event with the 100–200 m being the most frequent couple of disciplines (with a prevalence of 40–50%), and also, the 200–400 m couple was chosen by 20% of athletes.

## Figures and Tables

**Table 1:** Descriptive statistics and ANOVA outcomes.

		Men				
		N = 116	N = 89	N = 88	N = 26	N = 174
		Only 100 m	100 and 200 m	Only 200 m	200 and 400 m	Only 400 m
100 m	Age of peak performance	23.94 ± 3.02	24.37 ± 2.65			
	Peak performance	10.01 ± 0.07	9.99 ± 0.09			
200 m	Age of peak performance		23.78 ± 2.54	23.99 ± 2.88	24.27 ± 2.79	
	Peak performance		20.09 ± 0.23	20.20 ± 0.11 ^b^	20.14 ± 0.23	
400 m	Age of peak performance				24.12 ± 2.85	23.52 ± 2.58
	Peak performance				44.38 ± 0.56	44.82 ± 0.33 ^d^
		**Women**				
		**N = 88**	**N = 114**	**N = 60**	**N = 40**	**N = 159**
		**Only 100 m**	**100 and 200 m**	**Only 200 m**	**200 and 400 m**	**Only 400 m**
100 m	Age of peak performance	23.83 ± 2.78	24.32 ± 3.10			
	Peak performance	11.10 ± 0.10	11.01 ± 0.14 ^a^			
200 m	Age of peak performance		24.15 ± 3.11	23.08 ± 2.28 ^b^	24.73 ± 3.54 ^c^	
	Peak performance		22.43 ± 0.29	22.65 ± 0.18 ^b^	22.50 ± 0.28 ^c^	
400 m	Age of peak performance				24.13 ± 3.54	24.01 ± 3.02
	Peak performance				50.33 ± 0.72	50.94 ± 0.61 ^d^

Notes: ^a^ statistically significant difference in the only 100 m subgroup; ^b^ statistically significant difference in the 100 and 200 m subgroup; ^c^ statistically significant difference in the only 200 m subgroup; and ^d^ statistically significant difference in the 200 and 400 m subgroup.

## Data Availability

The data presented in this study are available on request from the corresponding author.
